# Photobiomodulation at Different Wavelengths Boosts Mitochondrial Redox Metabolism and Hemoglobin Oxygenation: *Lasers* vs. *Light-Emitting Diodes In Vivo*

**DOI:** 10.3390/metabo12020103

**Published:** 2022-01-23

**Authors:** Tyrell Pruitt, Caroline Carter, Xinlong Wang, Anqi Wu, Hanli Liu

**Affiliations:** 1 Department of Bioengineering, University of Texas at Arlington, 500 UTA Blvd, Arlington, TX 76019, USA; Tyrell.Pruitt@UTSouthwestern.edu (T.P.); caroline.carter@mavs.uta.edu (C.C.); xinlong.wang@uta.edu (X.W.); anqi.wu@uta.edu (A.W.); 2 UT Southwestern Medical Center, Department of Radiology, 5323 Harry Hines Blvd, Dallas, TX 75390, USA

**Keywords:** photobiomodulation, broadband near-infrared spectroscopy, oxygenated hemoglobin concentration, cytochrome c oxidase, redox metabolism, light-emitting diodes

## Abstract

Our group previously examined 8 min photobiomodulation (PBM) by 1064 nm laser on the human forearm in vivo to determine its significant effects on vascular hemodynamics and cytochrome c oxidase redox activity. Since PBM uses a wide array of wavelengths, in this paper, we investigated (i) whether different wavelengths of lasers induced different PBM effects, and (ii) if a light-emitting diode (LED) at a similar wavelength to a laser could induce similar PBM effects. A broadband near-infrared spectroscopy (bbNIRS) system was utilized to assess concentration changes in oxygenated hemoglobin (Δ[HbO]) and oxidized cytochrome c oxidase (Δ[oxCCO]) during and after PBM with lasers at 800 nm, 850 nm, and 1064 nm, as well as a LED at 810 nm. Two groups of 10 healthy participants were measured before, during, and after active and sham PBM on their forearms. All results were tested for significance using repeated measures ANOVA. Our results showed that (i) lasers at all three wavelengths enabled significant increases in Δ[HbO] and Δ[oxCCO] of the human forearm while the 1064 nm laser sustained the increases longer, and that (ii) the 810-nm LED with a moderate irradiance (≈135 mW/cm^2^) induced measurable and significant rises in Δ[HbO] and Δ[oxCCO] with respect to the sham stimulation on the human forearm.

## 1. Introduction

Photobiomodulation (PBM) uses lasers and/or light-emitting diodes (LEDs) to modulate cellular functions for physical or mental benefits. PBM has been investigated for years with animal models and human studies. For example, PBM was reported to be therapeutically beneficial for healing body wounds [1,2,3], reducing pains [4,5,6,7], treating specific brain injuries [8,9,10], and improving symptoms of depression [11,12,13]. In addition, when being applied to the forehead of healthy humans, PBM has proven its usefulness to promote attentive behavior, working memory, and executive functions [14,15,16,17,18]. 

While the exact mechanism of PBM is not entirely clear, one of the prevailing theories is that a photon is absorbed by the copper subunit of the terminal enzyme, cytochrome c oxidase (CCO), of the electron transport chains of mitochondria [19,20,21]. The light-absorbed CCO enhances the ability of the mitochondria to catalyze the reduction of oxygen to produce ATP more efficiently [22,23,24]. As CCO redox activity increases, oxygen consumption also increases, leading to a rise in the rate of oxidative phosphorylation [25,26] as well as cellular oxygen metabolism [27,28]. Since neurons have an increased reliance on mitochondrial oxygen metabolism compared to most other cell types, PBM has been shown to affect neuronal functions significantly [29,30,31]. However, this prevailing theory had not been evidenced by objective, physiological measures in human studies in vivo either during or after PBM until 2016, when we reported the first observation of PBM-induced enhancement of CCO redox activity and tissue vascular hemodynamics during and after 8 min PBM by 1064 nm laser in the human forearm [28]. 

In [28], we demonstrated that broadband near-infrared spectroscopy (bbNIRS), together with a non-linear regression algorithm, was the key means enabling us to quantitatively assess concentration increases of oxygenated hemoglobin, Δ[HbO]; deoxygenated hemoglobin, Δ[HHb]; and oxidized CCO or CCO redox state, Δ[oxCCO], of the human forearm in vivo during and after PBM. Furthermore, the technology of bbNIRS was validated with respect to MRS [32] and has been supported/utilized by numerous reports in human studies [32,33,34,35,36,37,38,39,40,41,42]. Thus, we reutilized it twice to quantify effects in vivo of Δ[HbO], Δ[HHb], and Δ[oxCCO] in the human prefrontal cortex stimulated non-invasively by the same 1064 nm laser on the human forehead, with high reproducibility [27,43]. 

Furthermore, light sources used in PBM devices are highly associated with the availability of lasers and LEDs in the market. Literature reviews of PBM have exhibited a wide range of wavelengths applied in both animal and human studies [44,45]. The most common wavelengths used in both lasers and LEDs are in the range of 600–900 nm [46], particularly at the three wavelengths of 660 nm, 810 nm, and 850 nm. The reason those wavelengths were chosen for PBM is because oxCCO has light absorption peaks at 660 and 800–850 nm [34]. Thus, these specific wavelengths enable oxCCO to be more stimulated with an increased concentration. On the other hand, 1064 nm laser or 1070 nm LEDs have demonstrated their effects on enhancement of human cognition [17,18,47,48]. Both 1064 nm and 1070 nm are not at the absorption peaks of oxCCO, but they have much less light scattering ability or a smaller light scattering coefficient than that at 800–850 nm light according to Mie theory [49,50]. Thus, 1064 nm or 1070 nm light can travel through deeper and more tissue volume and thus stimulate more oxCCO than 800–850 nm light. 

Quantitatively, our previous reports demonstrated significant enhancements of CCO redox metabolism by 1064 nm PBM in vivo [27,28,43]. It is unclear as to whether PBM by 800–850 nm laser or LED would create more or less effects than 1064 nm laser on CCO redox activity (i.e., Δ[oxCCO]) and vascular hemodynamics (i.e., Δ[HbO] and Δ[HHb). Such knowledge would exceptionally interest researchers, clinicians, and potential manufactures in the PBM field. A better understanding of PBM-induced enhancement on mitochondrial and hemodynamic activities by different wavelengths in human tissues in vivo would facilitate the optimal selection of wavelengths to achieve the best therapeutic outcomes for clinical use in the near future. 

Thus, in this study, we focused on PBM-induced effects by three wavelengths of lasers and a LED and quantified changes of CCO redox metabolism and vascular hemodynamics on the human forearm. Specifically, we utilized 800 nm, 850 nm, and 1064 nm lasers as well as an 810 nm LED to conduct the sham-controlled PBM experiments, following the same bbNIRS setup and protocol as previous studies [27,28,43]. In addition, the three lasers were set with similar power densities (or irradiance) for a fair comparison, while the LED’s irradiance was about 50% less than those of the lasers. Explicitly, this study would examine and support the following three working hypotheses:

**Hypothesis** **1.** *All three lasers at 800 nm, 850 nm, and 1064 nm promote similar PBM effects on Δ[HbO], Δ[HHb], and Δ[oxCCO] of the human forearm.*

**Hypothesis** **2.** *PBM-induced enhancements by the lasers at the three wavelengths on Δ[HbO] and Δ[oxCCO] of the human forearm are time- or dose-dependent on PBM, with different temporal patterns.*

**Hypothesis** **3.** *The 810 nm LED with a moderate power delivered on the human forearm creates adequate signals of Δ[HbO] and Δ[oxCCO], which are comparable to those by the 800 nm laser.*

## 2. Results

### 2.1. Optical Spectra of Lasers and LED and Laser-Induced Increases of Skin Temperature

**Figure 1:**
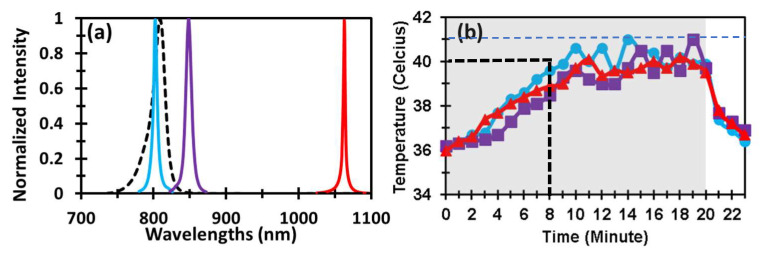
(**a**) Three normalized laser spectra at 800 nm (blue), 850 nm (purple), and 1064 nm (red), as well as one LED spectrum at 810 nm (dashed black curve), all of which were captured with the bbNIRS system. (**b**) It shows group-level (n = 4) temperature changes of the human forearm illuminated with 800 nm (blue), 850 nm (purple), and 1064 nm (red) lasers. The gray-shaded area indicates the 20 min active delivery of the lasers on the human forearm, while the last 3 min were during the post-PBM period. The black dashed vertical line at 8 min indicates the corresponding PBM period used in the study, which would raise the skin temperature no higher than 40 °C. The blue dashed horizontal line indicates the maximal skin temperature (≈41 °C) induced by the lasers.

Before examining PBM effects on the human forearm by different laser and LED wavelengths, we needed to obtain or assess their optical spectra with bbNIRS, followed by intensity normalization for spectral comparisons. Figure 1a shows respective normalized spectra with spectral peaks at 800 nm, 850 nm, and 1064 nm for the three lasers, as well as the LED peak at 810 nm. The values of full width at half maximum (FWHM) were 6 nm, 9 nm, and 3 nm for 800 nm, 850 nm, and 1064 nm laser, respectively, while the LED had a broader FWHM of 20 nm. It was clearly shown that the spectrum of 810 nm LED well covered that of an 800 nm laser. 

Furthermore, Figure 1b compares temperature rises induced by the three lasers. The temperature data were collected on the skin of four participants. Each curve in the figure represents a group-averaged (n = 4) temperature changes during the 20 min laser illumination. According to repeated measures ANOVA, no significant difference (*p* > 0.05) in temperature rise trajectory on the arm skin was observed among the three laser groups during the entire 23 min period with 20 min PBM and 3 min post-stimulation. This figure indicated that the thermal effect induced by each laser with a similar total power was consistent. This set of results helped us exclude/remove a confounding factor (thermal difference caused by different wavelengths) that may cause alteration in chromophore concentrations by other wavelengths. In addition, note that the maximal temperature rise by 8 min PBM at each of all three wavelengths was to be 4 °C or less (40 °C − 36 °C = 4 °C). Moreover, the skin temperature would remain below 41 °C, even when the lasers continued for another 12 min (gray-shaded area, 0–20 min).

### 2.2. PBM Effects by 1064 nm Laser on the Human Forearm 

**Figure 2:**
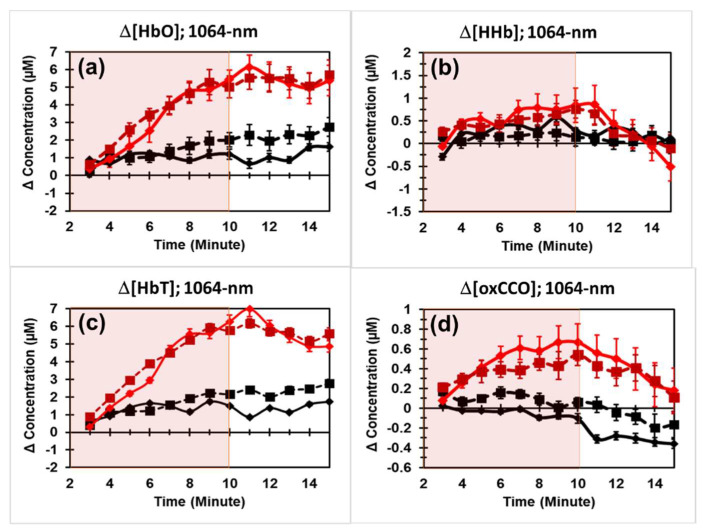
PBM-induced alterations of human forearm in (**a**) Δ[HbO], (**b**) Δ[HHb], (**c**) Δ[HbT], and (**d**) Δ[oxCCO] collected in a recent study (by solid curves), compared with the results (plotted by dashed curves) published in 2016 by Wang et al. [28]. In each panel, red curves and squares represent data under active PBM by 1064 nm laser; black curves and squares plot data under the sham condition. Error bars denote standard errors of the mean. Overall significance was tested using repeated measures ANOVA.

The newly acquired and quantified time series of Δ[HbO], Δ[HHb], Δ[HbT] (= Δ[HbO] + Δ[HHb]), and Δ[oxCCO] in response to 8 min PBM by 1064 nm laser are shown in Figure 2 (by solid curves in all four panels). It is unambiguous that all four time series exhibited clear and large increases under PBM conditions compared with those under sham conditions. Indeed, repeated measures ANOVA confirmed that all Δ[HbO], Δ[HHb], Δ[HbT], and Δ[oxCCO] had significant increases (*p* < 0.001) when compared to those under sham stimulation. 

Furthermore, to examine the reproducibility of our results, we also plotted our previous arm PBM results published in 2016 [28] in the same figure panels (by dashed curves). Highly similar trajectories are shown by visual inspection between the two datasets (i.e., collected in this study and the previous one) in each panel of Figure 2. More quantitative and rigorous statistical analysis by repeated measures ANOVA indicated that no significant difference (*p* > 0.05) existed between the two datasets for each of Δ[HbO], Δ[HHb], Δ[HbT], and Δ[oxCCO] changes under respective active and sham conditions. Given that these two datasets were collected four years apart with different spectrometers from different human participants by other experimental operators, the highly repeatable or reproducible results for forearm PBM by 1064 nm laser underscore the excellent robustness of the bbNIRS method and reliable or consistent physiological responses to PBM assessed by bbNIRS.

### 2.3. PBM Effects by 800 nm and 850 nm Lasers on the Human Forearm

**Figure 3:**
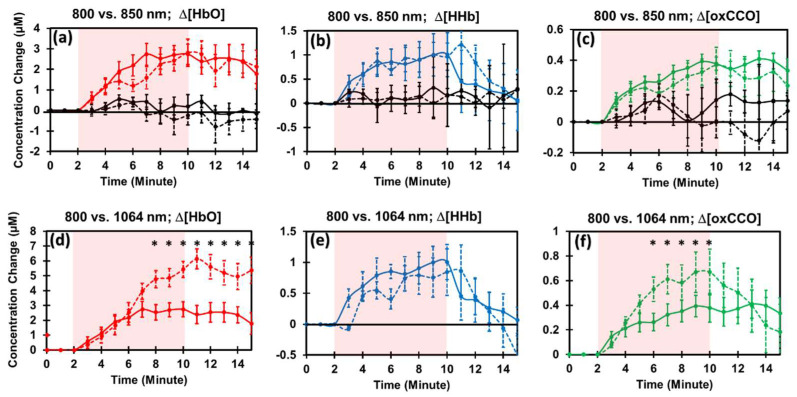
Group-averaged (n = 10) alterations in (**a**) Δ[HbO], (**b**) Δ[HHb], and (**c**) Δ[oxCCO] under sham (black curves) and laser stimulation (colored curves) at 800 nm (solid curves) and 850 nm (dashed curves). Stimulation epoch is marked with a pink-shaded block in each panel. Similarly, group-averaged (n = 10) changes in (**d**) Δ[HbO], (**e**) Δ[HHb], and (**f**) Δ[oxCCO] under 1064 nm laser (dashed curves) and 800 nm laser (solid curves). “*” indicates significant difference (*p* < 0.05) between each pair of (**d**) Δ[HbO] values and (**f**) Δ[oxCCO] values at each time point stimulated by the two lasers. This conclusion was derived from two-sample *t*-tests after completion of repeated measures ANOVA performed over the entire period of 8 min PBM and 5 min recovery for both Δ[HbO] and Δ[oxCCO].

Following the analysis procedures similar to Section 2.2, we assessed PBM effects by both 800 nm and 850 nm lasers on the human forearm. Figure 3a–c shows PBM-evoked increases of Δ[HbO], Δ[HHb], and Δ[oxCCO] as compared to those under sham conditions (by black curves) for 800 nm (solid) and 850 nm (dashed) laser illumination. Statistically, repeated measures ANOVA indicated that 8 min, 800 nm laser PBM induced significant enhancement (*p* < 0.001) in (a) Δ[HbO], (b) Δ[HHb], and (c) Δ[oxCCO] as compared to those by the sham stimulation. The same results were held statistically (*p* < 0.001) for the 850 nm laser PBM compared to its own sham conditions. Furthermore, highly similar trajectories between the two time series evoked by two lasers were clearly observed by visual inspection in Figure 3a–c. Consistently, repeated measures ANOVA confirmed that no significant (*p* > 0.05) difference existed between PBM-induced alterations by 800 nm and 850 nm lasers in each of the Δ[HbO], Δ[HHb], and Δ[oxCCO] cases.

### 2.4. Comparison of PBM Effects by 1064 nm vs. 800 nm Lasers

Since Section 2.3 showed that both 800 nm and 850 nm lasers would not significantly affect PBM effects on mitochondrial CCO activity and vascular hemodynamics of the human forearm, we next focused on finding the key and significant differences of PBM effects by 1064 nm and 800 nm lasers. Figure 3d–f compares the PBM effects evoked by these two lasers. We performed a two-step statistical analysis to identify critical differences in a dose-dependent manner. Analysis Step 1: Repeated measures ANOVA across the 13 min period (8 min PBM and 5 min recovery) reported that the 1064 nm laser stimulation created significant concentration growths in both Δ[HbO] (*p* < 0.001) and Δ[oxCCO] (*p* < 0.05) with respect to those by the 800 nm laser. This set of results made us pay special attention to only both Δ[HbO] and Δ[oxCCO]. Analysis Step 2: Two-sample tests were performed at each time point between each pair of Δ[HbO] values affected by the two lasers (see Figure 3d) and between each pair of Δ[oxCCO] values stimulated by the two lasers (see Figure 3f). The two-step statistical analysis justified that (i) both lasers did not introduce any significant difference in Δ[HbO] and Δ[oxCCO] in the first several minutes on the human forearm. (ii) Five minutes after the laser onset, increases in Δ[HbO] by 1064 nm laser became significantly higher than that by 800 nm laser, and this significant difference continued through the 5 min period after PBM (as marked in Figure 3d by “*”). (iii) Three minutes after the laser onset, a significant increase in Δ[oxCCO] by 1064 nm laser occurred and lasted until the end of PBM (see Figure 3f). 

### 2.5. PBM Effects by 810 nm LED and Comparison with Those by 800 nm Laser

**Figure 4:**
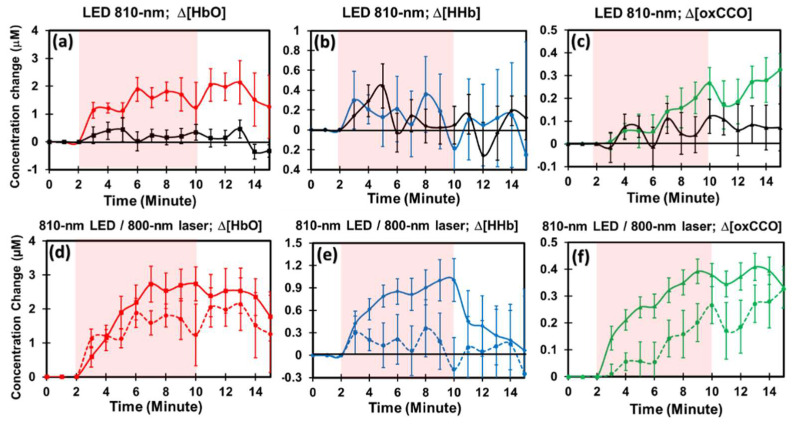
Group-averaged (n = 10) alterations in (**a**) Δ[HbO], (**b**) Δ[HHb], and (**c**) Δ[oxCCO] under sham (black curves) and 810 nm LED stimulation (colored curves). Stimulation epoch is marked with a pink-shaded block in each panel. Similarly, group-averaged (n = 10) changes in (**d**) Δ[HbO], (**e**) Δ[HHb], and (**f**) Δ[oxCCO] under 800 nm laser (solid curves) and 810 nm LED (dashed curves).

Figure 4a–c shows PBM-evoked increases by the 810 nm LED in Δ[HbO], Δ[HHb], and Δ[oxCCO] as compared to those under sham conditions (marked by black curves). Statistically, repeated measures ANOVA indicated that 8 min, 810 nm LED on the human forearm induced measurable and significant enhancement (*p* < 0.001) in (a) Δ[HbO] and (c) Δ[oxCCO] as compared to those by the sham stimulation. 

Furthermore, Figure 4d–f compares chromophore concentration changes caused by PBM with 810 nm LED and 800 nm laser on the human forearm. It was expected that all laser-produced changes in Δ[HbO], Δ[HHb], and Δ[oxCCO] were significantly higher than those stimulated by the LED because (1) the laser power density of the 800 nm laser (≈310 mW/cm^2^) was 2.3 times higher than that of the 810 nm LED (≈135 mW/cm^2^), and (2) the laser was much better collimated than the LED. The key messages learned from Figure 4d–f includes the fact that (1) a LED with a moderate power density (e.g., 135 mW/cm^2^) could generate comparable and proportional enhancement in both Δ[HbO] and Δ[oxCCO] with respect to those by a laser at a similar wavelength, and that (2) dose- or time-dependent increases promoted by the 810 nm LED and 800 nm laser followed a similar trajectory in both Δ[HbO] and Δ[oxCCO]. This set of observations helped us to have better confidence in LED clusters as PBM sources while their power densities are, in general, much weaker than those from lasers. 

## 3. Discussion

### 3.1. High Reproducibility of 1064 nm PBM on the Human Forearm 

As shown in Figure 2, the newly collected changes of Δ[HbO], Δ[Hb], Δ[HbT], and Δ[oxCCO] by 1064 nm laser PBM were highly reproducible with those previously reported results [28]. Note that the two sets of experiments were performed with two different bbNIRS systems by different operators from different human participants and taken several years apart. The fact that repeated measures ANOVA showed no significant difference between the recent and previous results for all four parameters convinced us that 1064 nm laser PBM on the human forearm over 8 min significantly increased hemodynamic oxygenation (i.e., Δ[HbO]), vascular blood volume (i.e., Δ[HbT] = Δ[HbO] + Δ[HHb]), and CCO redox metabolism (i.e., Δ[oxCCO]) as compared to the sham intervention. The high reproducibility of PBM effects on the forearm by 1064 nm laser demonstrated the robustness of the method and correctness of the results.

### 3.2. Experimental Evidence for Proval of Hypothesis 1 

Our results shown in Figure 3a–c illustrated clearly that both 800 nm and 850 nm lasers promoted identical PBM effects without any significant difference (*p* > 0.05) on Δ[HbO], Δ[HHb], and Δ[oxCCO] of the human forearm. Second, two lasers at 800 nm and 1064 nm exhibited very similar trends of PBM-induced rises in Δ[HbO], Δ[HHb], and Δ[oxCCO] (Figure 3d–f). All these observations supported our *Hypothesis 1*: *all three lasers at 800 nm, 850 nm, and 1064 nm promote similar PBM effects on* *Δ[HbO], Δ[HHb], and Δ[oxCCO] of the human forearm*. In other words, all three lasers at 800 nm, 850 nm, and 1064 nm were able to promote significant enhancement in hemodynamic oxygenation, vascular blood volume, and CCO redox metabolism. Moreover, 800 nm and 850 nm lasers produced identical, non-significant (*p* > 0.05) PBM effects on the human forearm in vivo in all three (or four) physiological metrics. This observation is expected since the light absorption and scattering properties of blood and CCO are very similar in the wavelength range between 800 and 850 nm. Thus, we would narrow our comparisons of PBM effects induced by 1064 nm and 800 nm laser (without 850 nm laser) in the following sub-sections.

### 3.3. Experimental Confirmation for Hypothesis 2

With close inspection on Figure 3d–f, we noted the time- or dose-dependent features in the three quantified metrics induced by the two lasers. Namely, the 1064 mm laser kept making gradual increases in Δ[HbO] and Δ[oxCCO] 5 and 3 min, respectively, after the onset of the laser. In contrast, the 800 nm laser maintained both Δ[HbO] and Δ[oxCCO] plateau during the last few minutes of PBM and 5 min post-stimulation. This observation implied that the 800 nm laser sustained the CCO redox metabolism and vascular oxygenation during the last few minutes of PBM and post-stimulation, while the 1064 nm laser was able to keep increases in CCO redox metabolism and vascular oxygenation during the later section of PBM. Moreover, Δ[oxCCO] by 1064 nm laser started to return to the baseline sooner than that by the 800 nm laser during the post-PBM period. All these observations confirmed our *Hypothesis 2*: *PBM-induced alterations by the lasers at the three wavelengths on Δ[HbO], Δ[HHb], and Δ[oxCCO] of the human forearm are time- or dose-dependent on PBM, with different temporal patterns.*

**Figure 5:**
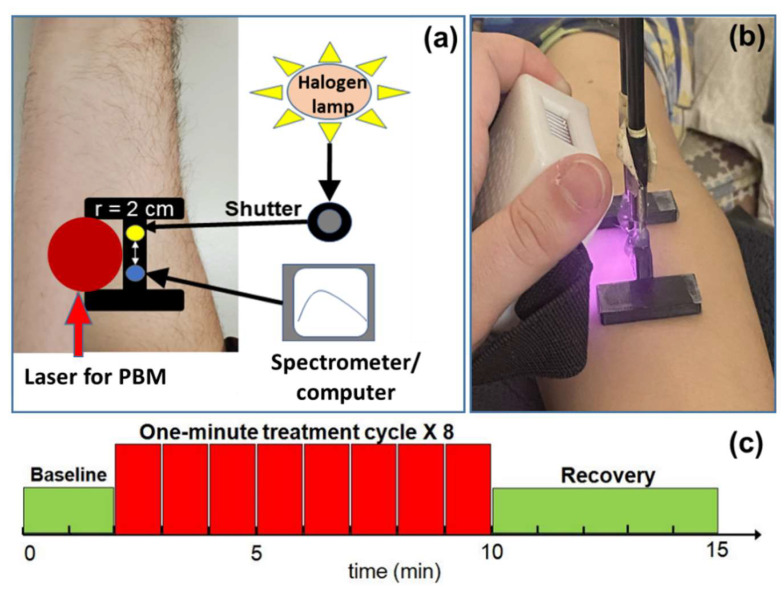
Experimental setup and protocol: (**a**) Schematic diagram for the bbNIRS system, consisting of (1) a black I-shape probe holder, which held 2 optical 3.5 mm fiber bundles with a center-to-center separation of 2 cm, (2) one fiber bundle (yellow) to a tungsten-halogen light source, and (3) another bundle (blue) to the QEPRO spectrometer connected to a laptop computer. The forearm PBM stimulation was administered through a 4.1 cm laser aperture (red circle). (**b**) A photo of the actual 2-bundle bbNIRS probe setup for data acquisition during LED-based PBM. The purple light is the 810 nm LED light invisible to our naked eyes but could be seen by a camera. (**c**) Experimental paradigm of the PBM/sham stimulation with an interleaved data collection arrangement. It contained one 2 min baseline (green), eight 1 min PBM/sham stimulation cycles (red) of 55 s PBM/sham and a 5 s bb-NIRS acquisition, and a 5 min recovery or post-PBM period.

The consistent dose-dependent features shown in Figure 3d,f imply that both 800 nm and 1064 nm lasers shared the same underlying mechanism of action for PBM in the initial 4 min period of PBM. The dose-dependent differences in Δ[oxCCO] and Δ[HbO] seen 3–5 min after the PBM onset can be attributed to three potential causes. First, one cause could result from the physical conditions of the lasers and measurement setup. Since the 800 nm laser was not well collimated, it attenuated laser irradiance more on the peripheral region (with a measured irradiance of 190 mW/cm^2^) than the 1064 nm laser (with a measured irradiance of 220 mW/cm^2^). Thus, changes in the redox activity and hemoglobin oxygenation by 800 nm PBM would be accumulatively less at the measurement site (see the setup in Figure 5a) than those by 1064 nm PBM. Second, light scattering was higher at 800 nm than at 1064 nm, so 1064 nm light can penetrate deeper into tissue. Thus, 1064 nm light can reach a deeper and broader volume of tissue for photo-oxidation, promoting more persistent or lasting rises in both Δ[oxCCO] and Δ[HbO]. Consequently, the 800 nm laser would interrogate a shallower and smaller tissue volume for PBM. As a result, a new equilibrium or balance between the increased mitochondrial metabolism vs. oxygen supplies would be achieved sooner than the 1064 nm laser. The 1064 nm laser maintained ongoing growth of Δ[oxCCO] and Δ[HbO] a few minutes longer. Finally, the last cause could stem from a physiological reason. Besides CCO, water has a higher absorption coefficient at 1064 nm than at 800 nm; 1064 nm laser may trigger other channels, such as transient receptor potential cation channel subfamily V member 1 (TRPV1) [51], leading to stimulations and increases of Δ[HbO] and Δ[oxCCO]. 

TRPV1 is often used in the body’s heat regulation and the sensing of heat [52], but it was also found to react to various other stimuli such as capsaicin [53], which are known to generate a sense of warmth. These TRPV1 channels have been called photothermal channels. However, we showed that the maximum skin temperatures induced by all three wavelengths were below or up to 41 °C (Figure 1b), below the supposed activation threshold of 43 °C [54], although partial opening is common in these gates at lower temperatures. 

### 3.4. Experimental Confirmation for Hypothesis 3

The 810 nm LED is the most commonly used light sources in the field of PBM. The main benefit of LED over laser stimulation includes low cost, safety, and ease of use [55]. However, due to the broader, weaker, and less focused nature of the LED light, it has been unclear as to whether LED-based PBM is similarly effective to laser-based PBM. In this study, we addressed this question by direct and quantifiable measurements in vivo and reported that 810 nm LED could significantly enhance Δ[HbO] and Δ[oxCCO] as compared to those under sham stimulation (see Figure 4a–c). Moreover, the results illustrated that the 810 nm LED PBM exhibited dose-dependent trends in both Δ[oxCCO] and Δ[HbO] similar to those by 800 nm laser. Since the irradiance of our 810 nm LED source was more than 50% weaker (i.e., 135 mW/cm^2^ at the center) than that of 800 nm laser (310 mW/cm^2^), PBM-induced effects on Δ[oxCCO] and Δ[HbO] by 810 nm LED would be smaller than those by 800 nm laser, as observed in Figure 4d,f. All these remarks strongly substantiated our Hypothesis 3: A 810-nm LED with a moderate power delivered on the human forearm creates adequate signals of Δ[HbO] and Δ[oxCCO], which are comparable to those by an 800 nm laser. 

The LED-based PBM results revealed three pieces of important experimental evidence: (1) an 810 nm LED was able to create significant stimulations on vascular hemodynamic oxygenation and CCO redox metabolism when the LED had a moderate irradiance (≈135 mW/cm^2^), regardless of its broader and non-focusing nature of light. (2) The dose-dependent trajectory by the 810 nm LED was similar to that by the 800 nm laser, hinting that both of them could result from the same mechanism of action because of the overlapping spectra of the two light sources (see Figure 1a). However, most commercial LED units have relatively much weaker irradiance [55] than that which we used in this study. Thus, prolonging the stimulation time to increase overall PBM dose would be an option. (3) It is intriguing to note that the LED-triggered increases in Δ[oxCCO] remained at the elevated level without a returning tendency at least during the 5 min post-PBM period. In comparison, this long-lasting effect of the boosted Δ[oxCCO] was also noted in the 800 nm laser case (Figure 4f). In contrast, the increased Δ[oxCCO] by the 1064 nm laser started returning to the baseline immediately after the cease of the laser. The underlying cause for 800 nm laser or 810 nm LED to be able to maintain the elevated CCO redox metabolism longer with respect to the 1064 nm PBM is still unclear. These novel and intriguing findings need to be first verified and then further explored in future studies.

### 3.5. Tool to Guide Light Selection and Dosage for Effective Clinical Applications of PBM

Besides increasing concentrations of oxidized CCO and oxygenated HbO, PBM produces transient reactive oxygen species (ROS). If elevated to a higher level for a long period of time, it can be detrimental to the cells [56]. However, transient increase of ROS is actually beneficial by upregulating enzymes that sequester reactive oxygen species. Moreover, it is known that the PBM dosimetry exhibits the dose–response phenomenon of hormesis, meaning that PBM leads to stimulation of a biological process at a low dose but inhibition of that process at a high dose [57]. Thus, the amount of metabolic stimulation by PBM should be carefully determined and controlled by the dosage and time of light illuminations to avoid extended production of ROS and the hermetic, biphasic effect in future implementation of PBM.

In Figure 2, Figure 3 and Figure 4, clear plateaus of Δ[HbO] and Δ[oxCCO] are shown in all the wavelengths of PBM, indicating that the benefits of PBM in hemodynamic and metabolic activations are time-dependent or dose-dependent. This extended period of plateaued increases in both [oxCCO] and [HbO] has the ability to provide many benefits due to increased ATP production [22] and an increased oxygenated hemoglobin supply [58]. On the basis of the findings in this paper, we demonstrated that bbNIRS is an excellent tool to facilitate quantification of PBM dosage for suitable delivery and beneficial effects.

### 3.6. Limitations of the Study and Future Work

While this study investigated PBM effects on the human forearm in vivo by 800 nm, 850 nm, and 1064 nm laser, as well as 810 nm LED, we recognized several limitations of the study. First, our sample size was relatively small (n = 10) for the laser and LED measurements. Along the same line, temperature data were collected on an even smaller population (n = 4), albeit for multiple measurements. Second, our three lasers did not have completely identical setup conditions, namely, laser irradiance and collimation conditions. These non-identical setups gave rise to the variability in tissue volume stimulated or interrogated by light sources with different uniformity and in detected signal intensities, leading to inconsistent PBM effects measured by bbNIRS. Third, with only a 2 cm source–detector separation, bbNIRS was more sensitive within 1 cm tissue depth to detect PBM effects. Since 1064 nm light is scattered less within tissue, it penetrates tissue deeper than 800 nm and 850 nm light. Thus, the detection sensitivity of bbNIRS would be wavelength dependent. Third, bbNIRS was quite sensitive to movement noise, and thus a small sample size might have amplified the variance among individuals. Fourth, it is known that PBM is limited by the penetration depth because of light absorption and scattering in the human tissue and brain. Thus, PBM is not proper for deep tissue/brain stimulations. 

The PBM research on physiological effects quantitatively measured in controlled human studies in vivo is in its early phase. For future work, many mechanistic questions need to be answered before PBM can become an effective intervention tool for clinical applications. Moreover, it is critical to develop and achieve a quantitative dose–response relationship for PBM to be applied in humans (such as the brain) in order to avoid extended production of ROS and the hermetic, biphasic effect. 

## 4. Materials and Methods

### 4.1. Participants

Two groups of healthy normal subjects were recruited for the study. The first group of 10 participants (6 males and 4 females; Group 1; 21–35 years of age) was measured for the 1064 nm laser PBM on the right forearm. The second group of 10 healthy human subjects (5 males, 5 females; Group 2; 8 being 21–35 years of age and 2 being 60–65 years of age) participated in the forearm PBM measurements with 800 nm and 850 nm lasers as well as with an 810 nm LED. All the participants were recruited from the University of Texas at Arlington and were screened for eligibility before acceptance into the study. 

Exclusion criteria of participants included anyone who (1) took any medication or drug for vascular circulations, (2) was pregnant (self-report), (3) had any history of arm injury or arm surgery in the last 12 months, (4) had neuropathy or skin numbness, and (5) was a diabetic patient as required by the manufacturer of the laser (Cell Gen Therapeutics LLC, Dallas, TX, USA). In addition, all the participants were told to avoid any caffeine beverages 2–3 h before each experiment. For Group 1, all eligible participants underwent the active and sham experiments, in a random order, with 1064 nm laser given on the right forearm several days apart to ensure no pre-treatment effect. For Group 2, each eligible participant had three separate forearm PBM visits with 800 nm laser, 850 nm laser, and 810 nm LED delivered on the right forearm, and respective sham given on the left forearm. We ensured a 1 week rest between any two visits for all participants in Group 2. The study was approved by the institutional review board at the University of Texas at Arlington and complied with all applicable federal NIH guidelines. Before all experiments, informed consent was obtained from each participant. 

### 4.2. Instrumentation for PBM and bbNIRS

Similar to the protocol used in our previous study [28], sham and active PBM were administered with a continuous-wave (CW) 1064 nm laser provided by Cell Gen Therapeutics LLC, Dallas, TX (Model CG-5000), which is FDA-cleared for use on humans for relief of pain. During sham stimulation, the laser was set to a minimum power of 0.1 W and blocked with a black cover to prevent any residual laser light from reaching the participant’s forearm. On the other hand, 800 nm and 850 nm CW lasers were acquired from Changchun New Industries (Optoelectronics Tech. Co., Changchun, China), while a LED unit at 810 nm was custom built with a single LED light unit. For the 1064 nm laser, the irradiance (power density) was set to be 250 mW/cm^2^ across the well-collimated laser beam of 4.1 cm in diameter. For the 800 nm and 850 nm lasers, the peak irradiances at the center of the 4.1 cm diameter laser beams were 310 and 330 mW/cm^2^, respectively, since these two lasers were not fully collimated with the irradiances on 1 cm peripheral regions of the beams to be approximately ≈190 and 210 mW/cm^2^. These laser irradiance values were frequently checked and confirmed with a power meter before each measurement. Furthermore, the maximum irradiance from the LED unit was ≈135 mW/cm^2^, which was used for the LED-based PBM experiments on the human forearm.

Furthermore, a single channel bbNIRS system was used to measure concentration changes in several chromophores *in vivo.* As shown in Figure 5a, the system consisted of a tungsten-halogen lamp (Model 3900, Illumination Technologies Inc., East Syracuse, NY, USA) as the light source and a sensitive CCD-array spectrometer (QEPRO, Ocean Optics Inc., Orlando, FL, USA) as the light detector. The source and detector fibers were set 2 cm apart via 3.5 mm optical fiber bundles in a 3D-printed probe holder [28]. Note that the source-detector probe was placed as close as possible to the tissue under PBM (see Figure 5a) without blocking the laser beam for acquiring the largest or most significant effects of PBM. However, since the light from our LED unit was not collimated, it expanded greatly in a 1 cm distance with a much larger area than that by any of the laser beams (see Figure 5b). The probe holder was affixed to the participant’s arm using a piece of double-sided tape to minimize motion artifacts caused by any movement of the subject. The broadband light was diffused through the forearm tissue and then acquired by the spectrometer via the detection fiber bundle during the data acquisition period.

### 4.3. Experimental Setup and Protocol

Both sham and active PBM experiments on the human forearm were conducted in a locked room with all reflective surfaces removed. No external windows were present to pollute the spectrum. A warning sign indicating a laser was in use inside was also used to prevent individuals without goggles from entering. Protective goggles (900–1000 nm: 5+, 1000–2400 nm: 7+; 2900–10,600 nm: 7+) were worn by everyone present in the room at all times. Participants were instructed to close their eyes during the experimental protocols for added eye protection and blinding to the type of PBM stimulation being given on a particular day. The probe holder was placed in roughly the same location on each participant’s forearm for each visit. 

The experimental protocol consisted of a 2 min baseline, 8 cycles of 55-s PBM and 5 s bbNIRS acquisition, and a 5 min post-stimulation (PBM or sham) measurement (Figure 5c). Because of potential light contamination or interference, we used an interleaved protocol between laser/LED in PBM and the white light source in bbNIRS (see Figure 5c). Specifically, our PBM laser was set up to pause laser light after every 55 s delivery by an internal shutter while the laser itself was still on. As soon as the active or sham PBM light paused, the shutter for the white light was manually open, and bbNIRS immediately acquired data for 5 s continuously (i.e., integration time = 5 s). The time delay between pausing the laser light and beginning the bbNIRS measurement was less than 1 s. All the data collected during this 5 s period were included and averaged. The same interleaved PBM/recording protocol (55 s PBM and 5 s recording) was repeated 8 times/cycles for PBM/sham periods. The data acquisition during the baseline and recovery followed the same style for consistency. A total of 15 data points/spectra were collected for each subject. The 2 min baseline spectra were used as the reference point for subsequent concentration changes of Δ[HbO], Δ[HHb], Δ[HbT] (=Δ[HbO] + Δ[HHb]), and Δ[oxCCO]. 

Participants received no information as to which laser or sham protocol they would receive on an experimental visit. Laser/sham equipment was always set up after the participant was instructed to close their eyes, ensuring proper blinding. 

### 4.4. Spectra of Lasers/LED and PBM-Induced Temperature Changes

Since we investigated PBM effects on the human forearm by lasers at three wavelengths and one LED unit, it was necessary to compare their spectral peaks and widths. To do so, we measured and then normalized the respective optical spectra of three lasers and one LED unit by collecting the light from each of the optical sources with the same CCD-array spectrometer (QEPro, Ocean Optics Inc.). 

Furthermore, a change in skin temperature caused by different lasers could result in alteration in Δ[HbO], Δ[oxCCO], and other parameters. Thus, temperature measurements using a handheld infrared thermometer (Medical Head and Ear Thermometer, Metene, Shenzhen, China) were performed on a smaller group of subjects (n = 4) to examine whether different wavelengths of lasers would increase the forearm skin temperatures differently. The area or the spot location that the temperature was measured would be near the central stimulation region where the sensing port/nozzle of the thermometer pointed to. While the spot size of the temperature sensing could not be quantified, we expected that it should be smaller than or at least within the light-stimulation area. The thermal recording was taken during the 5 s laser shuttered periods, similar to the bbNIRS measurements with the same interleaved fashion, except with 1 min baseline, 20 min laser PBM, and 3 min recovery for each of the three lasers at 800, 850, and 1064 nm. Data collection for thermal readings occurred during the 5 s, PBM-off periods throughout the entire 24 min experimental period.

### 4.5. Data Processing and Statistical Analysis

The non-linear, curve-fitting regression algorithm previously developed in Wang et al. [28] was followed this study to quantify PBM-induced changes in chromophore concentrations of the human forearm, namely, Δ[HbO], Δ[HHb], Δ[HbT], and Δ[oxCCO]. Specifically, captured experimental spectra were fitted in the range of 750–900 nm on the basis of the modified Beer–Lambert law. Multiple wavelengths in such a broad spectral range facilitated more accurate values for the fitted parameters. 

For statistical analyses, we first tested if treatment (PBM vs. sham) and time caused variation in each chromophore concentration (i.e., Δ[HbO], Δ[HHb], Δ[HbT], and Δ[oxCCO]). When the time effect was significant, it means that the effect of PBM was time-varying. Both treatment and time aspects could be tested at once using repeated measures ANOVA [59,60]. This set of ANOVAs was repeated for comparison between laser versus sham conditions under each of the three laser PBM and under 810 nm LED/sham condition throughout the 8 min intervention and 5 min recovery period. The same ANOVA was also performed to compare changes of each chromophore concentration induced by different PBM wavelengths or by laser versus LED across the 13 time points. Second, two-sample tests were taken at each time point between active and sham measurements for each chromophore concentration (i.e., Δ[HbO], Δ[HHb], and Δ[oxCCO]) [61] if the repeated measures ANOVA showed significant differences between active and sham PBM conditions. Third, the same type of repeated measures ANOVA [59,60] was performed when comparing (1) the current 1064 nm forearm PBM results vs. those previously reported in [28], and (2) temperature changes induced by three lasers at respective wavelengths. Two-tailed *p* < 0.05 was considered significant. Last, individual subjects were tested against the group-level mean using the interquartile rule method to determine if removal from a particular dataset was necessary as an outlier from the true mean. 

## 5. Conclusions

This study demonstrated the high reproducibility of PBM-induced effects by 1064 nm laser on CCO redox metabolism and hemoglobin oxygenation of the human forearm unambiguously by performing a rigorous statistical analysis (i.e., repeated measures ANOVA) between the newly versus previously collected data. These well reproducible results reinforce the statement that bbNIRS is a reliable method and enables quantitative assessments of PBM effects on human tissues *in vivo*. 

This study also examined PBM-induced effects by three wavelengths of lasers with comparable irradiance and by a LED at a similar wavelength to a laser. The results made us draw three conclusions. First, all three lasers at 800, 850, and 1064 nm enabled dose-dependent, significant stimulation or enhancement of mitochondrial redox activity, vascular oxygenation, and vascular blood volume and flow of the human forearm. Second, the 1064 nm laser sustained longer and more increases of the physiological effects as compared to the other two lasers. The 1064 nm PBM could be attributed to (i) the well-collimated beam, (ii) deeper penetration depth because of weaker scattering effects, and (iii) other unknown light-absorbing sources boosting CCO redox metabolism. Third, the 810-nm LED significantly boosted CCO redox metabolism and vascular oxygenation with a similar trajectory (but smaller amplitude) to the 800 nm laser. In this case, the LED had an irradiance (≈135 mW/cm^2^ close to the LED emission unit) less than 50% of that (≈310 mW/cm^2^ near the laser center) from the laser. This conclusion may hint and support that safer, medium-powered, LED units or clusters may take an important role in the future PBM field. However, note that our conclusions need to be verified with a larger sample size or a more subject pool before anyone makes decisions on selections of wavelengths and between lasers vs. LED for future PBM devices. 

## Data Availability

The data presented in this study are available on request from the corresponding author—because we have not setup a public archive platform for data sharing.
